# TiO_2_–Ag–NP adhesive photocatalytic films able to disinfect living indoor spaces with a straightforward approach

**DOI:** 10.1038/s41598-023-31464-4

**Published:** 2023-03-14

**Authors:** Salvatore Chirumbolo, Davide Gibellini, Luca Berto, Cinzia Cirrito, Antonio Vella, Geir Bjørklund, Andrea Sbarbati, Paolo Bernardi, Umberto Tirelli

**Affiliations:** 1 Department of Neurosciences, Biomedicine and Movement Sciences, Unit of Human Anatomy, University of Verona, Strada Le Grazie 8, 37134 Verona, Italy; 2 Department of Diagnostics and Public Health, Unit of Microbiology, University of Verona, Verona, Italy; 3 Material Chemical Expert Labs, Treviso, Italy; 4 Tirelli Medical Group, Pordenone, Italy; 5 Azienda Ospedaliera Universitaria Integrata, Verona, Italy; 6 Council for Nutritional and Environmental Medicine, Mo i Rana, Norway

**Keywords:** Biological techniques, Biotechnology, Microbiology, Environmental sciences, Health care, Health occupations, Materials science

## Abstract

TiO_2_–Ag doped nanoparticulate (TiO_2_–Ag–NP) adhesive photocatalytic films were used to assess the ability in dropping down the burden of indoor microbial particles. The application of an easy-to use photocatalytic adhesive film to cleanse indoor living spaces from microbial pollution, represents a novelty in the field of photocatalytic devices. Reduction was attained by photocatalysis in selected spaces, usually with overcrowding (≥ 3 individuals) in the common working daily hours, and upon indoor microclimate monitoring. TiO_2_–Ag doped nanoparticulate (TiO_2_–Ag–NP) adhesive photocatalytic films were applied within five types of living spaces, including schools and job places. The microbial pollution was assessed at time 0 (far from routine clean, ≥ 9 h) and throughout 2–4 weeks following the photocatalyst application by relative light unit (RLU) luminometry and microbial indirect assessment (colony forming units per cubic meter, CFU/m^3^). TiO_2_–Ag–NP photocatalyst reduced RLU and CFU/m^3^ by rates higher than 70% leading to RLU ≤ 20 and microbial presence ≤ 35 CFU/m^3^. The described TiO_2_–Ag–NP is able to reduce microbial pollution to the lowest RLU threshold (≤ 20) within 60 min in open daylight in a standardized test room of 100 m^2^. The correlation between RLU and CFU/m^3^ was positive (r = 0.5545, *p* < 0.05), assessing that the microbial reduction of indoor areas by the TiO_2_–Ag–NP adhesive film was real. Titania photocatalysts represent promising tools to ensure air cleaning and sanitization in living indoor microclimates with a low cost, feasible and straightforward approach. This approach represents an easy to handle, cost effective, feasible and efficacious approach to reduce microbial pollution in indoor spaces, by simply attaching a TiO_2_–Ag–NP adhesive film on the wall.

## Introduction

The use of titanium dioxide with Ag nanoparticulate thin films (TiO_2_–Ag–NP) as a photo-oxidative catalyst to remove chemical pollutants or microbial contamination, dates back to few decades ago, when this approach was appreciated for its cost effectiveness, highest oxidation rate at room temperature, high duct velocities and low pressure drop tolerance^1–6^.

Different kinds of thin film technologies, such as spin coating^7^, e-beam evaporation, chemical vapor deposition^8^ or magnetron sputtering^9^, are able to build up a composed thin film of TiO_2_ elements (100 nm) joined and/or complexed with silver (Ag) nanoparticles (usually ≤ 10 nm, range 1–100 nm), via various methods, such as doping^10,11^, heterojunction formation^12^ or metal ion implantation or others^13^. The component TiO_2_ works as a semiconductor, having an energy gap (EG) = 3–3.3 eV, despite this value depends on the different allotropic forms of titania. When TiO_2_ is irradiated with photons of an energy amount greater than EG (i.e., corresponding to a wavelength, λ ≤ 390 nm), then an electron is able to overcome this energy gap and can be promoted from the valence band to the one of charge conduction. The valence gap is able to react with the absorbed water molecules upon the thin film of the photocatalyst, forming some oxygen-derived radicals, such as the hydroxyl radical (·OH^-^), which are able to greatly damage bacteria cells. or directly upon any adsorbed organic compounds. Then, the photocatalytic film on an adhesive support^14,15^, is able to convert oxygen reactive species (ROS) to hydrogen peroxide, which is used by Ag to enhance bacterial killing^16^.

To date, the widespread application of devices using TiO_2_–Ag nanotechnology is rapidly growing up, both in public indoor spaces and household facilities, because of an increasing acknowledgment towards the TiO_2_–Ag–NP ability in reducing indoor microbial contamination and leaving a microbe-free environment^17–19^. The use of newly patented TiO_2_–Ag–NP photocatalytic films^19,20^, enabled to be easily applied to walls or windows in order to exert a fine clearance of the airborne microbial pollution, is attracting public interest due to the relative low cost, ease to handle and low toxicity of TiO_2_-mediated photocatalysis^20^. We recently demonstrated that these easy-to-handle photocatalytic devices (thin adhesive films) were able to drop down the microbial pollutants in the indoor spaces of public vehicles, usually overcrowded with pupils, students or customers^20^. This study represents a further assessment of our research in indoor living spaces.

Actually, the evidence here described represents a novelty in the field of photocatalytic materials used to clean indoor environments from airborne microbial particles. Despite the use of TiO_2_ photocatalysis for indoor environments dates back to nineties, yet regarding chemical pollution (volatile organic compounds), scant attention is devoted to the use of TiO_2_–Ag–NP to reduce microbial contamination in an indoor living space, as chemical pollutants still represent the major targets of photocatalysis application^1,21^. The technological challenge is to plan an easy-to-handle photocatalyst to be applied in any indoor space in an eco-sustainability manner. The ability of our photocatalytic adhesive films to cleanse human indoor spaces, is crucial if devices are particularly easy to handle, cost effective, hard-wearing and highly effective in reducing microbial and chemical pollution in a living space, such as schools or work places, in a widely feasible way. The photocatalytic film here described is very easy to be applied in indoor environments to reduce airborne microbial pollution, it works once simply sticking the adhesive film on indoor vertical enlightened surfaces.

Furthermore, the recent study by Matsura and colleagues, confirmed by others, assessed that TiO_2_-mediated photocatalysis, i.e., a titanium dioxide photocatalyst-coated glass film, exhibited the ability to inactivate within 20 min 99.9% of SARS-CoV_2_ in aerosol, by destroying virus particles and their genomes^19,22,23^. The ability of titania photocatalysts to exert a sanitizing action in indoor climates is widely known and particularly topical.

Actually, photocatalysis with titanium dioxide materials is emerging as a novel, straightforward technology to reduce microbial contamination in indoor environments^20,24^. The interest in the so-called photocatalytic disinfection has come to an exponential increase in recent years, exceeding 800 reports in the field^24^, probably due to a marked improvement in the many photocatalytic technologies for indoor sanitization^20,24–26^. Some indoor environments are particularly crucial for safety concerns. Besides healthcare structures and hospices, schools and educational institutes, usually crowded with children, represent a fundamental issue of ensuring a safe living space, as children and teenagers too, are considered at risk category subjects^28–30^. The same may be held for daily workers, to ensure a healthy indoor living and reduce job-related injuries.

In this context, the ability of our TiO_2_–Ag–NP photocatalyst to reduce microbial particles in an indoor environment may be considered an important technological advancement.

The purpose of this research study is to evaluate the ability of a hi-tech straightforward TiO_2_–Ag–NP photocatalyst nanotechnology, arranged in a simple adhesive film, to drastically reduce the airborne microbial content in indoor environments, so to generate a clean, disinfected and healthy space in which stably living, studying or working.

## Methods

### Selection of indoor spaces and microenvironment assessment

Major eligibility criteria for spaces selection in our study referred to previously published reports in order to assess a quite constant microclimate in our indoor environments^21–33,35^, so to significantly reduce the impact of outliers, bias and confounders in our collected data. To extend our investigation to the widest typology of indoor spaces undergoing our photocatalyst technology, we selected: (a) four different school classrooms (data collection from May 25th 2021 to June 7th 2021); (b) a farm showroom (data collection from April 14th 2021 to June 14th 2021); (c) two different housing farm boxes (job containers) (data collection from April 29th 2021 to May 13th 2021); (d) a laundering depot in a laundry (data collection from June 11th 2021 to June 16th 2021); (e) a phone shop (data collection from September 16th 2021 to November 12th 2021). Applications of two WIWELL TiO_2_–Ag–NP dimensional types, i.e., Type-1 (0.6 m × 0.9 m, = 0.54 m^2^) and Type-2 (0.3 m × 0.5 m, = 0.15 m^2^), were applied within the indoor spaces, taking care to not use a single photocatalytic film and allow a wide spreading of more available films to detect homogeneously all the indoor environment. Due to the different macro-environment conditions (the relationship of the building with outside climatic parameters) it was difficult to standardize an exact number of WIWELL TiO_2_–Ag–NP films, therefore, on the basis of previous experience^20^, we arranged the photocatalytic films as follows. Any school classroom was 6 m width × 10 m length × 5 m height (60 m^2^) and was equipped with a total 1.62 m^2^ WIWELL TiO_2_–Ag–NP n. 3 Type-1 adhesive photocatalytic films. The farm showroom was 10 m length × 10 m width × 3.5 m height (100 m^2^) and was equipped with a total 2.16 m^2^ WIWELL TiO_2_-Ag-NP n. 4 Type-1 adhesive photocatalytic film. Any housing farm box was 19.95 m^2^ and 69.85 m^3^ (WIWELL TiO_2_–Ag–NP n.4 Type-2 adhesive photocatalytic film = 0.6 m^2^). The laundry depot was 12 m^2^ (42 m^3^), whereas the phone shop was 60 m^2^ (300 m^3^). WIWELL TiO_2_–Ag–NP adhesive photocatalytic films = 0.6 m^2^ and 1.62 m^2^ were applied respectively.

Microclimate evaluation in these different indoor spaces were not significantly affected by the different seasonal periods to gather raw data, as sampling were collected by preventing microclimate transient differences between indoor and outdoor environments, in order to set the thermal difference of ambient air and the difference in relative humidity small enough to resemble a steady-state (difference in the Universal Thermal Index, ∆UTCI, ≤ 0.5 °C)^34^. Eligibility indoor climate criteria for data inclusion in the study were: a) temperature range 21–26°C (69.8–78.8 °F), relative humidity (RH) 40–60%, CO_2_ between 250 and 1000 ppm; PM_2.5_ ≤ 12 μg/m^3^, ventilation and thermal comfort following the European standard EN 16,798:2019 and further evaluations^35^, light exposure as described further on.

### Sampling, TiO_2_–Ag–NP handling and data management

Samples were collected by swab methods^36^. Each swabbing was performed far from the routine cleaning settings (≥ 9 h from chemical cleansing) by one of ours (Luca Berto) alongside with one assistant (Antonio Vella).

**Figure 1:**
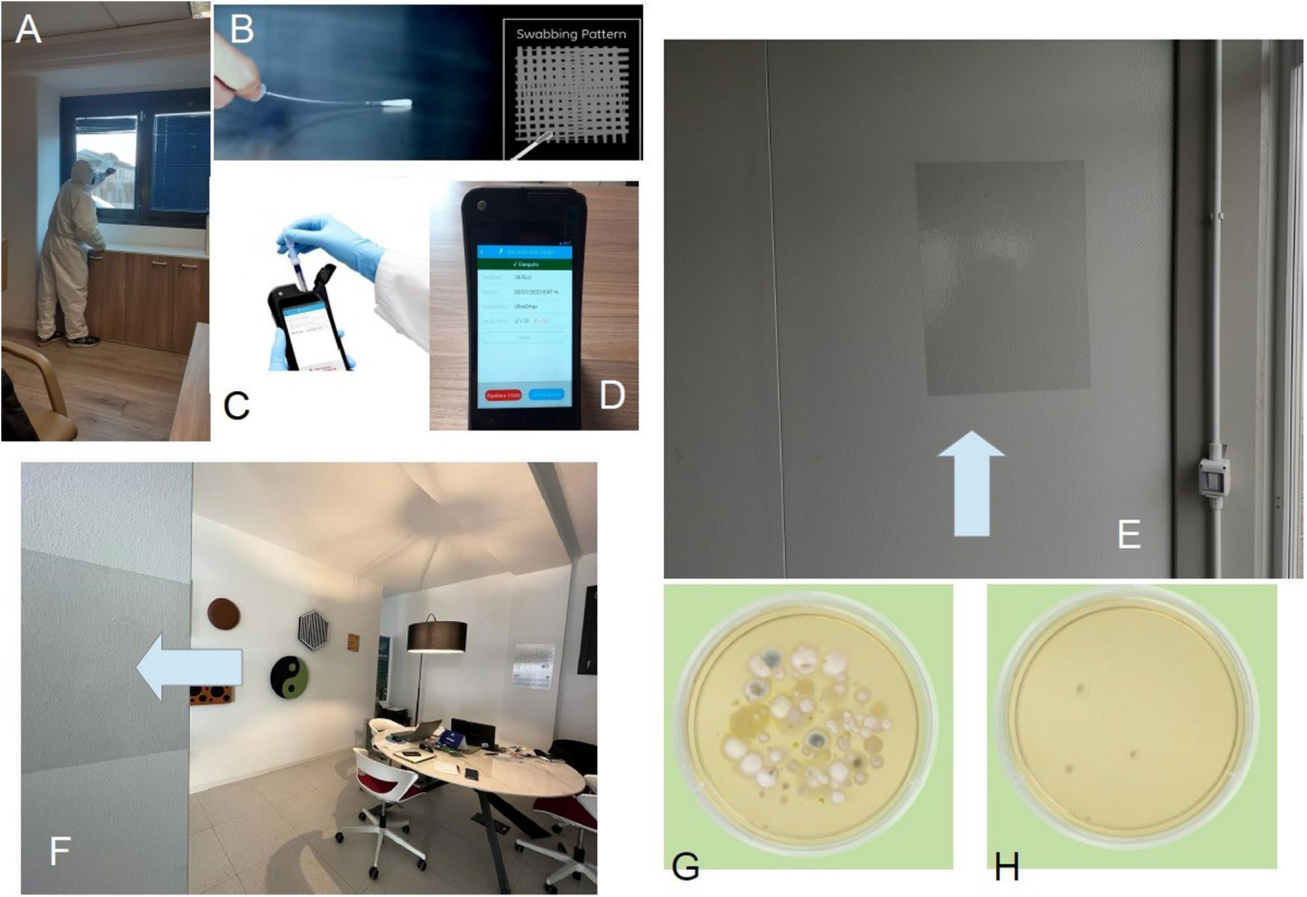
Brief summary of the process of swab test for RLU performance. (**A**) The co-author LB while swabbing on the inside part of a vertical surface (a window glass, for example), (**B**) Way by which the swab must be handled and used on the surface; (**C**) Inserting the swab in the RLU detection device; (**D**) an example of RLU read out; (**E**) and (**F**) Different examples of the WIWELL TiO_2_–Ag–NP application (a job container and an office); (**G**) Microbiology output on a TSA plate (37 °C, 48 h) before and (**H**) after the application for 12 h of the WIWELL TiO_2_–Ag–NP adhesive film.

Figure 1 summarizes the process of sampling by swab method.

Initially, the operator leaves UltraSnap swab be equilibrated to room temperature (about 21–25 °C) before any intended use, then he holds the swab firmly and, by twisting and pulling top of the swab out of its tube, makes it ready to use it. Subsequently, he thoroughly swabs a standard area (Fig. 1A) following the producer’s instruction and swabbing a 4 × 4 inches or 10 × 10 cm area, following a zig zag path (Fig. 1B). Areas (surfaces) are usually inner part of windows (glass) or indoor objects far from human contacts. The operator replaces the swab into the swab tube, then he uses thumb and forefinger to break the Snap-Valve, holding firmly the tube and bending the bulb backward and forward for some seconds. He therefore squeezes the bulb at least twice to allow the liquid expulsion down the swab shaft, shakes the swab bud in the liquid for 5–10 s and then he will read the sample in the bio-luminometer (Fig. 1C) within 30 s, holding luminometer upright. Then he presses the starting button and will read the RLU value (Fig. 1D).

Sampling was performed in quadruplicate, i.e., for each evaluated site at least 4 swabs were performed in the same sampling collection (each sample = 4 swabs) and all performed starting at 3.00 p.m. ± 15 min, far from routine cleansing time (6.00 a.m.). Each swab was carried out in an empty space without persons, except for testing performers, where the complete absence of crowding people was induced at least 30 min before collection, in order to standardize the indoor climate parameters and prevent statistical confounders. Each tested indoor space was endowed with one simple type of WIWELL TiO_2_–Ag–NP photocatalytic film, attached on different walls, either on a supporting frame or directly, 20 h before the sampling. The TiO_2_–Ag–NP photocatalyst is an adhesive sheet with two dimensional typologies, as reported above: (a) a Type-1 (60 × 90 cm) sheet (used for experiments in schools and work indoor places), (b) a Type-2 (30 × 50) cm sheet in farm housing cabs. Each photocatalyst, appearing as an adhesive panel on the wall, was set at a distance from the center of the room ≤ 3.00 m, from the floor ≥ 1.5 m, from the top ≥ 1.0 m. The photocatalyst was left on its place for all the experimental time.

Sampling was performed at 3.00 p.m. in the day before the experiment and before attaching the WIWELL TiO_2_–Ag–NP photocatalytic film (control samples) and in the day/days of experiment, with the previously attached WIWELL TiO_2_–Ag–NP photocatalytic film (data samples). The number of sampling data for each indoor investigated area was calculated by sample size statistics in order to achieve a margin of error close to 5%. Therefore, a number of at least 70 samples was considered sufficient to reach a 95% of a population proportion undergoing the effect with a 95% confidence interval (error = 5.11%). Data from time 0 and the end of data collection were independently elaborated. The Cohen k for the accordance (89.74%) was 0.6533.

Sampling spots were designed in order to include at least four indoor sub-areas and eight sampling options, depending on the study rationale, from the photocatalyst source and ventilation, within 1.5 m (near), i.e., near/far the photocatalyst, near/far a window or a door, near/far the center of a room, near/far the walls of a room. As data regarded the complessive indoor volume of the investigated room, spots did not differ each other significantly, whereas outliers (≥ 3 SD) removed from the final data managing.

### Swabbing for luminometer evaluation and RLU calculation

The evaluation of adenosine triphosphate (ATP) was carried out exclusively by trained personnel.

A surface of about 100 cm^2^ (10 × 10 cm) was thoroughly swabbed using an ATP-swab following two opposite swabbing directions. Any ATP sampling was performed with ATP-swabs provided by the manufacturer (Ultrasnap-ATP monitoring Hygiena). Samples were all indifferently performed while the indoor space was lighted by daily sunlight, in a range from 1000 lx, which typically identifies an overcast day in midday, and 120,000 lx (brightest sunlight) at the same time (3.00 p.m. ± 15 min), depending on local seasonality and/or weather, except for the experiments on photocatalyst’s performance (see below). We previously demonstrated that the photocatalytic property of the WIWELL TiO_2_–Ag–NP did not change significantly upon different light sources^20^.

Following sampling, swabs were put in a manual calibrated and automatic bio-luminometer (ENSURE Touch, HYGIENA Ultrasnap model), enabled to provide an immediate and timely output into Relative Light Units (RLU), usually within 15 s and with a sensitivity up to 0.1 femtomoles of ATP for sample. The device is calibrated to give as positive control the β-light calibration range 270–330 RLU and a negative control at background (blank) of 0–5 RLU. The amount of light emission from the luminescent probe is expressed as RLU respect to a calibrated standard and is linearly dose-dependent from the concentration of microbial ATP^37^.

RLU breakthrough values < 20 indicated a clean surface, whereas values ≥ 60 RLU represented a dirty minimal threshold, i.e., assessing a not cleaned surface following at least one hour of direct contact with humans. Therefore, a target reduction of RLU ≤ 20 RLU or 70% to control, represented our primary end point.

### Performance evaluation of the WIWELL TiO_2_–Ag–NP photocatalytic adhesive film

A RLU separate experiment, involving six different indoor brightness conditions, was also considered in order to evaluate the performance of the WIWELL TiO_2_–Ag–NP adhesive film in different photocatalysis contexts: (a) direct sunlight (30–100 × 10^3^ lx); (b) ambient daylight (10–25 × 10^3^ lx); (c) sunset or sunrise (typically 400–50 lx); (d) overcast daylight (1,000 lx); (e) indoor ambient light 1 (750 lx); (f) indoor ambient light 2 (250 lx). Indoor brightness was measured with a Luxmeter PCE-VDL 16I (PCE Instruments, GmbH, Germany) and reported in lux. The WIWELL TiO_2_–Ag–NP adhesive films (4 Type-1 films) were applied within the indoor space at least 24 h before the test start. No chemical cleanse was performed in the 24 h before and throughout the experimental run. Each of the six different conditions were performed in six different days. Swabbing was executed on the same test indoor environment (a thermal insulated room 10 × 10 × 3.5 m, 100 m^2^, 350 m^3^, 55–60% relative humidity, 0.07–0.25 m/sec indoor ventilation) on 5 different swab spots in duplicate every 10 min to 1 h and at 90, 120, 150, 180 and 240 min. The assay was performed by two operators (LB, AV), in order to properly arrange and manage the time course test, according to the RLU evaluation described before.

### Indoor air spread microbial counting in colony forming units per cubic meter (CFU/m^3^)

In order to verify the environmental conditions of airborne bio-pollutants, such as bacteria or fungi, air sampling was carried out using a specific portable sampler instrument for microbiological control (MICROFLOW ALPHA, Aquaria, Italy), according to UNI-EN ISO 14,698:2003, EN 17,141:2020 and UNICHEM method 1962–2^38,39^. The instrument allows an auto-calibration control, via the automatic calibration system developed by the Polytechnic University of Milan (Italy) (report n.377/2003) and certified by INRIM in Turin (Certificate n.10-0114-01/2010).

For routine microbiological analysis a 90 head mm model (380 filtering holes 1 mm) was charged with a 90 mm ICRplus Tryptic Soy Agar (TSA) plus Lecitin-Tween 80-Histidine and Thiosulfate (TSA + LTHTh) culture gamma-irradiated plates (Merck-Millipore, Darmstadt, Germany) for each test, in order to investigate the environment presence of indoor ambient microbes. The plates allowed to incubate aerobic, microaerophilic and anaerobic bacteria. To investigate also the presence of yeasts and molds a Sabouraud Dextrose contact Agar + LTHTh culture medium was used in at least four separate occasions.

A total volume sample of 200 L (100 L/min for 2 min) was captured and filtered by the device and analyzed according to previously published methods^40^. Plates were incubated at 35 °C for 24 h (bacteria) and 48 h (yeasts and molds). Moreover, to evaluate the impact of microbes’ sizes for breathing areas, a TCR TECORA Pollution Check IMP-6 Bio (6 stages aerodynamic particle sizing) was used in a 100 m^2^ test room at 28.3 L/min sampling flow. The instrument was equipped with 90 mm TSA plates. The microbiologist evaluated the presence of different microbes according to at least six different decreasing stages, based on microbial known dimensions: (1) > 7.0 μm; (2) from 7.0 to 4.7 μm; (3) from 4.7 to 3.3 μm; (4) from 3.3 to 2.1 μm; (5) from 2.1 to 1.1 μm; (6) from 1.1 to 0.65 μm. Stages reflects the location of depositing of inhalable bacteria in the human lungs, where 1–2 altogether represents bacteria of the upper airway and 3–6 the respirable microbial particles^40,41^. Collection was performed in the so-called human breathing zone, considered at 1.5 m above the floor and 1.0 m from the walls. Microbes were counted as colony forming units per cubic meter (CFU/m^3^) as mesophilic microbial load at 36 °C, according to the ISO 4833–1: 2013, ISO 13,138:2012 standardized method^40^. Static sedimentation sampling on plate count and potato dextrose (PD) static agar plates was also performed.

### TiO_2_–Ag–NP film biomaterial and toxicologic profile

The TiO_2_–Ag–NP adhesive film is produced by WIWELL, Polcenigo (PN, Italy) and is made by a mixture of the photocatalyst titanium dioxide (TiO_2_), doped with Ag nanoparticulates (WIWELL), on a polyvinyl-elastomer adhesive film (WIGLASS), having different producer’s dimensions, yet the optimal ratio [indoor area/TiO_2_–Ag–NP surface] should be ≥ 40 (e.g., a 1.5 m^2^ TiO_2_–Ag–NP film for a 60 m^2^ room, approximately, considering an average height of 2.5–3.0 m).

The material is considered safe, from a toxicological point of view. Despite some controversial reports^42,43^, few data about the toxicology of titanium photocatalysts showed that TiO_2_ may be neurotoxic at 2.5 mg/kg body weight (bulk TiO_2_) in experimental animals^44^, yet the TiO_2_ EC_50_ is about 5.83 mg/L^45^ and moreover toxicological tests performed on *S. cerevisiae* reported no toxicological action even at 20,000 mg/L^46^, values that should ensure for any toxicological impact of possible titanium leakage from the WIWELL nanoparticulate^20^.

As regarding silver nanoparticles (Ag–NPs) toxicity in the photocatalytic membrane, the calculated EC_50_ is 2.0 μg/L for Ag–NP ≤ 5 nm^47^, and, as percentage dissolution of Ag ions has been reported to be ≤ 0.26% (i.e., 0.13–0.26%)^48^, considering that Ag is from 0.72 to 6.75% weight of the photocatalyst, the Ag leakage from a WIWELL membrane should be approximately ≤ 1.0 μg/L^49^.

The optimal efficiency of the TiO_2_-Ag-NP photocatalytic action (> 99%) can be reached at a luminosity higher than 109,000 lx under direct sunlight, i.e., during the brightest sunlight exposure (120,000 lx) or bright sunlight (111,000 lx) but the actual efficiency of any bacterial removal (> 99.99%) is achieved by simply 90 min of activation at 2000 lx, therefore even during a complete overcast midday, as over 90% of UV-A rays can pass through clouds and glass windows^20^.

### Optical and SEM imaging of the TiO_2_–Ag–NP photocatalytic film

Optical imaging was obtained with an Olympus BX51 light microscope. Very small samples 9 × 9 mm (thickness of 2–3 mm) of the photocatalytic film were set on aluminum stubs endowed with sticky carbon, then sputter coated with an ultrathin layer of colloidal gold and observed with an Environmental Scanning Electron Microscope (ESEM) (ESEM XL 30 FEI Philips low vacuum).

### Statistics

Data were collected as mean ± standard deviation (SD) or medians. These latter were also assessed in their distribution by a Mood’s test and a Sign test, at *p* < 0.05. Sample size test and Cohen k were considered for sample statistics. Distribution of data and normality were assessed with a (Kolmogorov–Smirnov (KS) test and a Lilliefors’ test. Pearson correlation at *p* < 0.,05 was performed. Analysis of variance with a two tailed paired t -test was performed in order to analyze data. Statistics were calculated with a SPSS v 24 software and plotted with Smart Statistics v.11 software.

## Results

### Reduction of RLU

**Figure 2:**
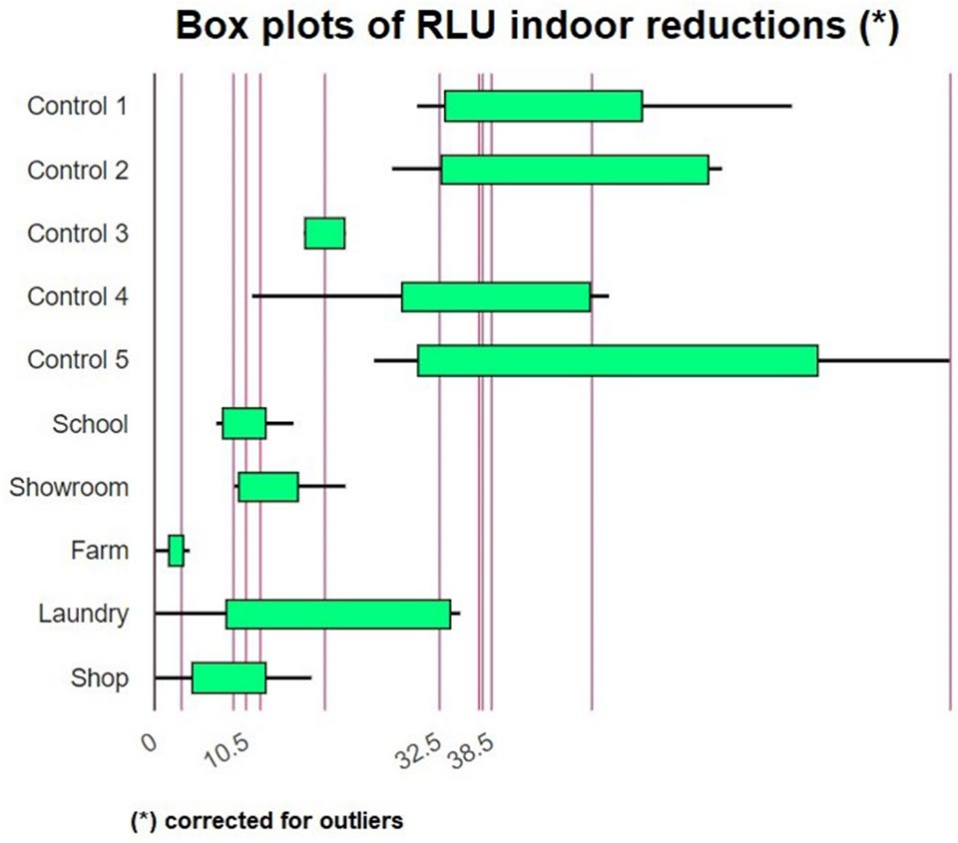
Box plot with means and medians (limits by 95% interval confidence) of the RLU values for controls (evaluations before the application of the TiO_2_-Ag-NP) and data averaged and estimated at the end of the collection. See text for details.

Figure 2 shows that WIWELL TiO_2_–Ag–NP photocatalysts are able to significantly reduce the Relative Light Units (RLU) evaluated in different sampling spots (quadruplicate replicates) within 5 different indoor spaces respect to controls. Table 1 shows the two-tailed sign test for each median statistic:

**Table 1:** Median test for data reported in Fig. 1

Indoor place	Median sampling	Median controls	Z score	*p*
School	56	13	2.4495	0.0143
Showroom	62	11	2.2361	0.0253
Farm	19.5	3	2.2358	0.0254
Laundry	38.5	32.5	1.6399	0.1025
Shop	37	12	3.0000	0.0027

The ability of TiO2–Ag–NP, to reduce microbial contamination as evaluated by RLU, was significantly reported for the majority of the evaluated indoor spaces, with the exception of laundries (*p* > 0.05), where it is widely known that laundering operations may represent a significant source of microbial carry over^50^.

**Figure 3:**
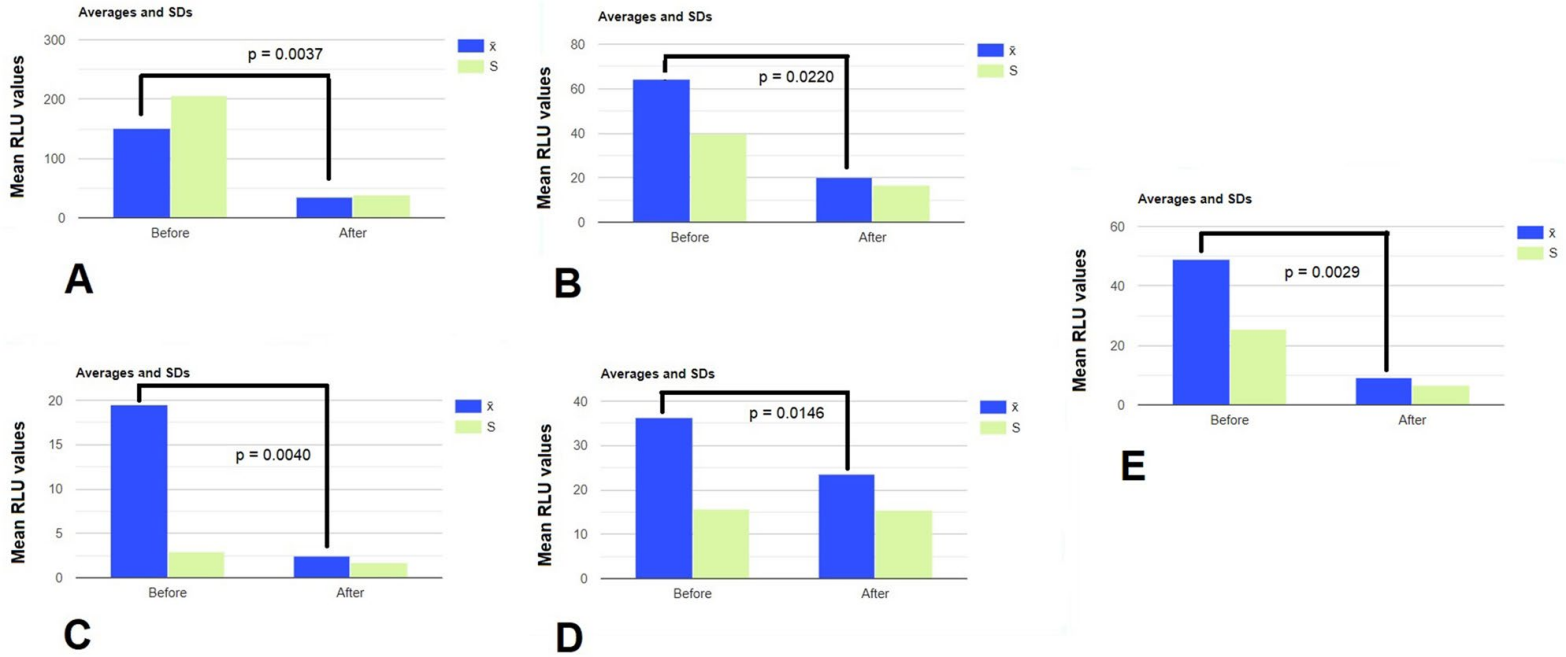
Means (blue) ± standard deviation (SD) (light green) of the RLU values (control vs TiO_2_–Ag–NP treated areas for each investigated indoor place: (**A**): school; (**B**): farm showroom; (**C**): farm housing cabs; (**D**): laundry; (**E**): shop. Statistics with Kolmogorov–Smirnov (KS) test and two tailed paired t-test at *p* < 0.05.

Figure 3 shows the reduction in RLU for any single indoor environment selected for the study.

RLU average data for the indoor space A (Fig. 2A) were non-normally distributed (Lilliefors’ test *p* = 0.0002985, K = 1.1982, skewness (S) = 2.9534) and showed a single outlier (RLU = 558), which, once removed, allowed the distribution to report a reduced skewness (S = 1.7767), yet significantly non-parametric (*p* = 0.04912). Following the application of one single type of TiO_2_–Ag–NP adhesive film (60 × 90 cm) in the tested indoor space on May 25th 2021 and collecting data throughout 12 days (June 7th 2021), the average value of RLU from 6 different sampling spots within the lived indoor area, dropped down from 150.33 ± 20.61 SD to 39.02 ± 5.33 SD (= − 74.04%) (Fig. 3A). When we applied the TiO_2_–Ag–NP at the wall of a wider showroom, we monitored the microbial reduction any week on 5 sample spots for 2 months (from April 14th 2021 to June 17th 2021) and reported a mean RLU reduction from 64.20 ± 9.60 SD to 20.20 ± 6.93 SD (= − 68.53%, *p* = 0.02201), above to the usual routine cleaning procedures (Fig. 3B). A significant reduction in RLU values was also achieved by applying the TiO_2_–Ag–NP (smaller dimensions) within two industrial housing modules for 2 weeks. Figure 3C shows that, despite the previously cleaning process, the TiO_2_–Ag–NP maintained longer and increased the sanitized indoor environment reducing RLU by about 87.18% (from 19.5 ± 2.88 SD to 2.5 ± 1.73 SD, *p* = 0.004056).

In areas where laundering processes are carried out, e.g., dirty linen storage, the effectiveness of TiO_2_–Ag–NP appeared somehow less warranted (Fig. 3D). Data of average RLU were normally distributed (Lilliefors’ test *p* = 0.1284, K = 0.7467, S = − 0.5626), On the contrary, the application of the TiO_2_–Ag–NP in the indoor space of a phone shop reported, following a 2-months monitoring (from September 16th 2021 to November 12th 2021) on 9 sample spots, an RLU reduction from 48.88 ± 25.21 SD to 9.33 ± 6.76 SD (= − 80.91%, *p* = 0.0029) (Fig. 3E).

**Figure 4:**
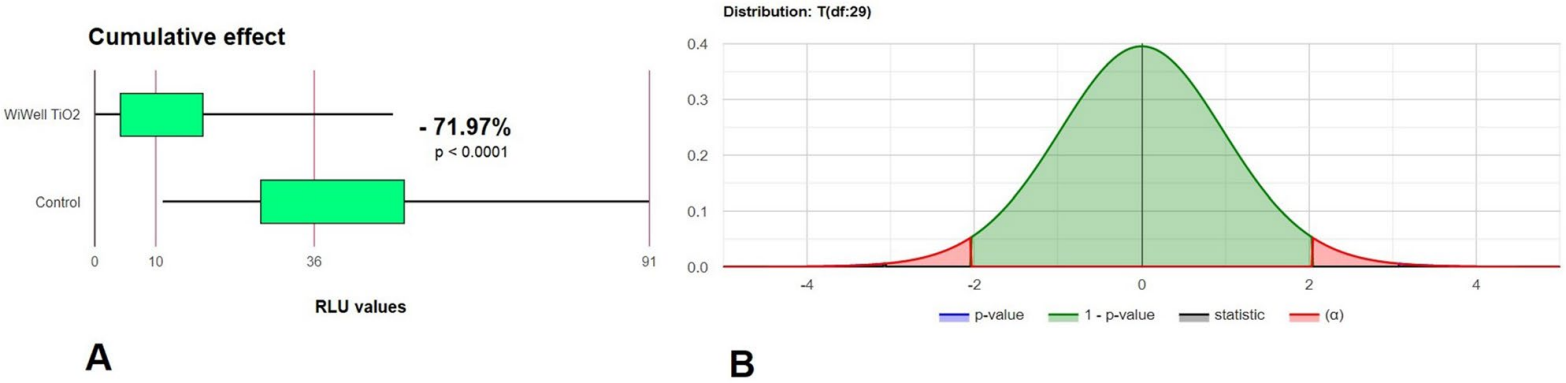
Cumulative data of the difference in RLU between controls and TiO_2_–Ag–NP treated areas (**A**) with its Lilliefors’ distribution as *p* values (**B**).

Taken as a whole, the application of the WIWELL TiO_2_–Ag–NP reduced the RLU by 71.97% (*p* < 0.0001) (Fig. 4A), whereas the probability distribution of samples is shown in Fig. 4B. Data distribution of the whole collected RLU values used in the study, subjected to the Lilliefors’ test, showed a marked difference from normality (*p* = 1.187 × 10^–13^, K = 2.2347, S = 5.8125), with positive asymmetry and kurtosis (excess kurtosis = 39.2901). Removal of outliers restored the normal distribution (Lilliefors’ test, *p* = 0.07143).

**Figure 5:**
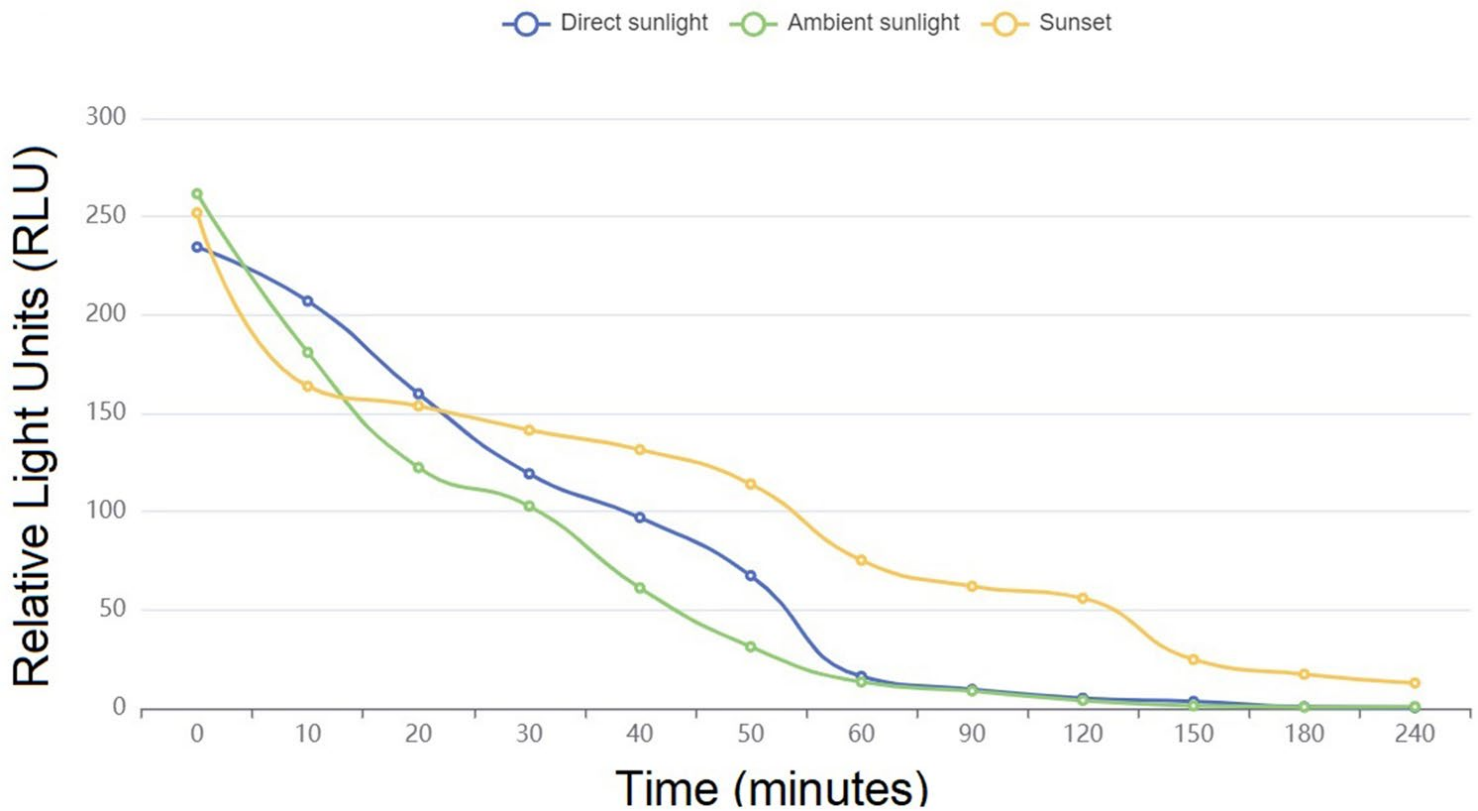
Time course of the WIWELL TiO_2_–Ag–NP photocatalytic film performance, evaluated in RLU, upon different daylight exposures: blue line (direct sunlight 60,000 lx); green line (ambient daylight 15,000 lx); orange line (sunset 100 lx). Plotting with Smart Statistics v.11 software.

**Figure 6:**
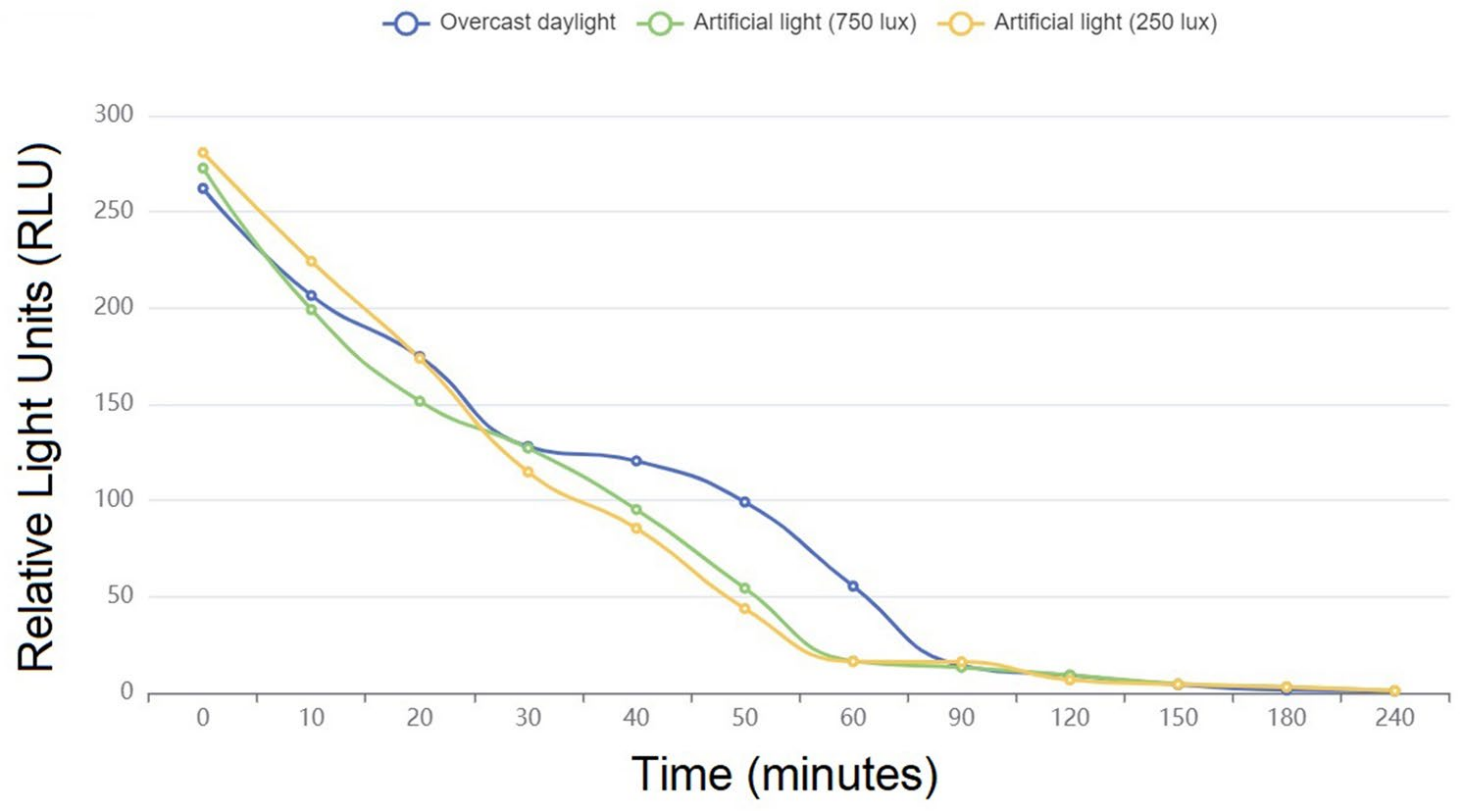
Time course of the WIWELL TiO_2_–Ag–NP photocatalytic film performance, evaluated in RLU, upon different ambient and artificial light exposures: blue line (overcast daylight 1000 lx); green line (artificial lamp, 750 lx); orange line (artificial lamp, 250 lx). Plotting with Smart Statistics v.11 software.

Figure 5 describes the time course of RLU decrease when the WIWELL TiO_2_–Ag–NP works in open daylight (without artificial lamps). The photocatalytic film reaches its lower threshold (≤ 20 RLU) within the first 60 min exposure, whereas during sunset at a luminometry ≤ 100 lx, this value is achieved by 120 min continuous exposure (Table 2). On artificial light emission (Fig. 6), the photocatalysis is perfectly working, dropping down the RLU value within the first 60 min exposure (see also Table 2). Anyway, an overcast daylight appeared less efficient (90 min instead of 60 min, Table 2) than an artificial light, probably due to irradiance differences. Usually, during an overcast daylight, artificial light can be switched on in offices and work places, so this may be not a real concern for the photocatalyst.

**Table 2:** Performance of the WIWELL TiO_2_–Ag–NP photocatalytic film upon different light sources.

Condition	Times (minutes)				
	0	60	90	120 or beyond	*p*
Direct sunlight 32–100 × 10^3^ lx	234.50 ± 16.89	16.25 ± 1.91 SD	< 20	< 20	0.00090686
Ambient daylight 10–25 × 10^3^ lx	261.60 ± 17.40	13.40 ± 2.27	< 20	< 20	0.00016211
Sunset or sunrise 400–50 lx	251.90 ± 6.66	75.30 ± 2.26	62.00 ± 2.36	17.30 ± 1.42	0.00016876
Overcast daylight 1000 lx	262.20 ± 7.31	55.40 ± 1.95	14.20 ± 1.15	< 20	0.00016305
Ambient 1 750 lx	272.70 ± 23.92	16.50 ± 2.07	< 20	< 20	0.00017462
Ambient 2 250 lx	280.80 ± 6.20	16.30 ± 2.26	< 20	< 20	0.00017360

### Reduction of CFU/m^3^

The effect of the WIWELL TiO_2_–Ag–NP in dropping down airborne microbes from the indoor environment has been evaluated also via the direct investigation of the microbial growth on agar plates and calculating the Colony Forming Units for cubic metre of air (CFU/m^3^)^49^. No bacterial and moulds growth was observed on PD static agar plates (if we except two cases/10 total) when microbial ambient content dropped down to ≤ 20 RLU (an example in Fig. 1H). The results of the TCR TECORA Pollution Check IMP-6 Bio (6 stages aerodynamic particle sizing) showed that microbial contamination was manly ≤ 5 CFU/m^3^ in the range 5–7 μm.

**Figure 7:**
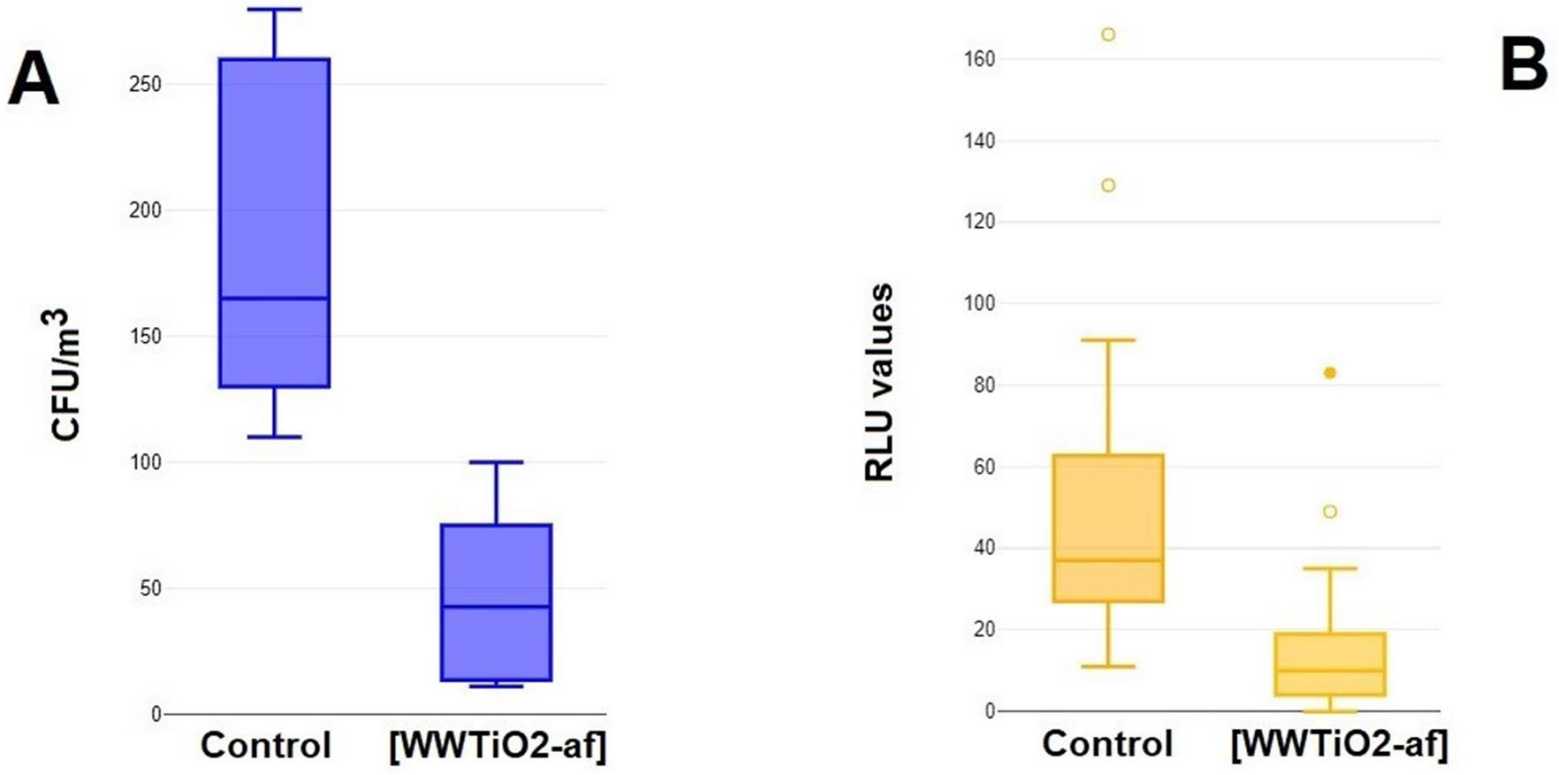
Comparison between reduction of microbial pollution in CFU/m^3^ (**A**) and RLU (**B**) in a cumulative data of 83 data points. See text for details.

Figure 7A shows that the reduction in CFU/m^3^, due to the application of the TiO_2_–Ag–NP film in indoor living environments, was as low as 74% (− 74.38%, *p* = 0.0003892), from 182 ± 62.50 SD CFU/m^3^ to 46.62 ± 35.30 SD. Overall data exhibited a normal distribution (Lilliefors’ test *p* = 0.7882, K = 0.4789, S = 0.4557), yet the existence of two outliers in the TiO_2_-Ag-NP sampling group (90 and 100 CFU/m^3^), when removed, leads the mean value below 35 CFU/m^3^ (30.50 ± 22.06 SD). Figure 7B summarizes the overall difference before (control) and following the application of the WIWELL TiO_2_–Ag–NP product, in order to compare the TiO_2_-membrane effect on air microbial pollution (two outliers were removed in Fig. 7B). Pearson’s test was adopted to assess the comparison between two fundamental methods, an indirect and more precise (RLU) and a direct and more accurate (CFU/m^3^) one, to assess the reduction of microbial population in the indoor spaces endowed with the photocatalytic device.

**Figure 8:**
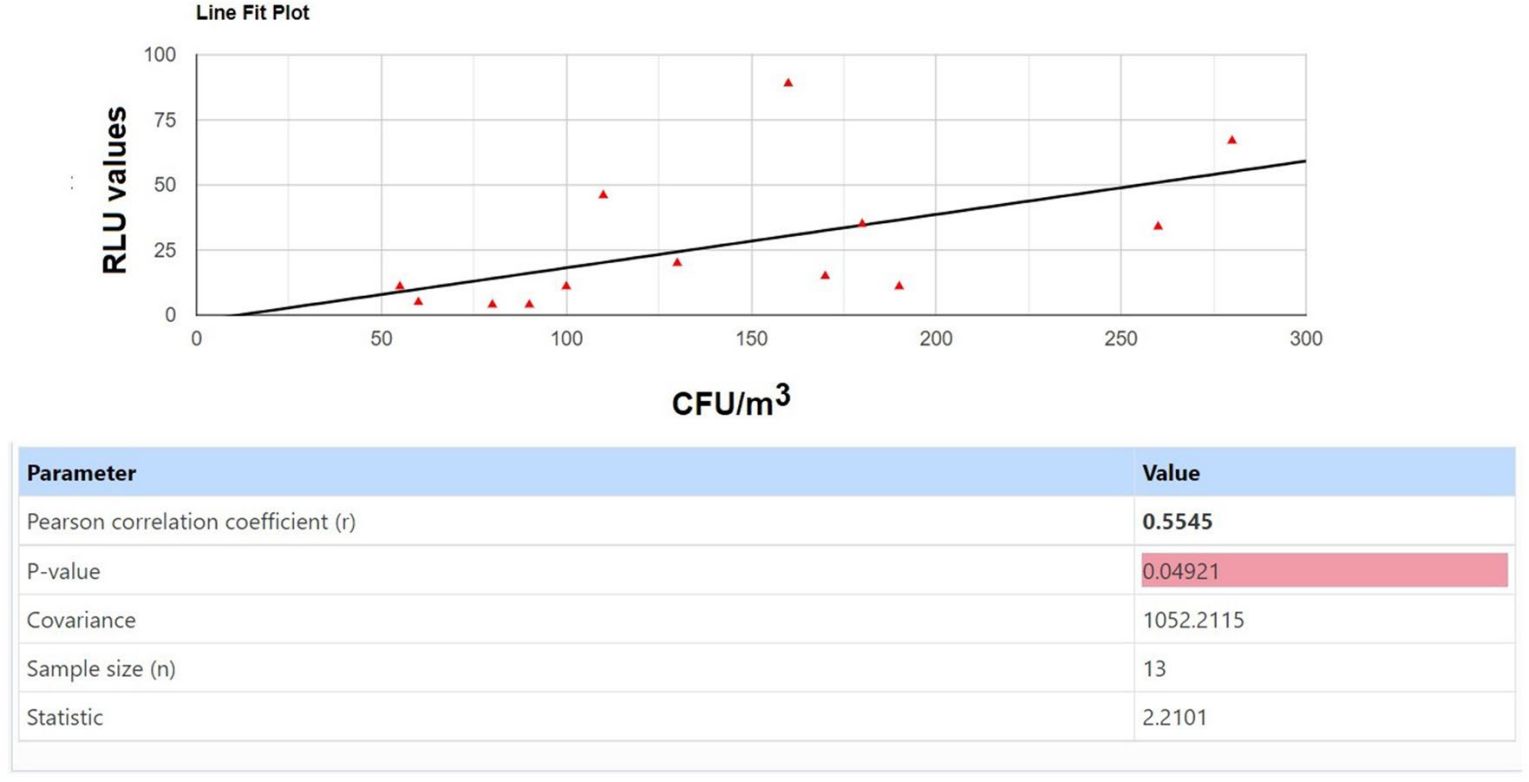
Pearson correlation of data collected for Fig. 4 by matching the relative delta between controls and TiO_2_–Ag–NP treated areas. See text for details.

Pearson’s correlation test between the percentage of microbial reduction by TiO_2_–Ag–NP calculated either in RLU or in CFU/m^3^, reported a positive correlation (r = 0.977, *p* = 0.04921), so assessing that both analytical evaluations assessed correctly the reduction of indoor microbial pollution by the TiO_2_-Ag-NP photocatalytic adhesive film (Fig. 8).

**Figure 9:**
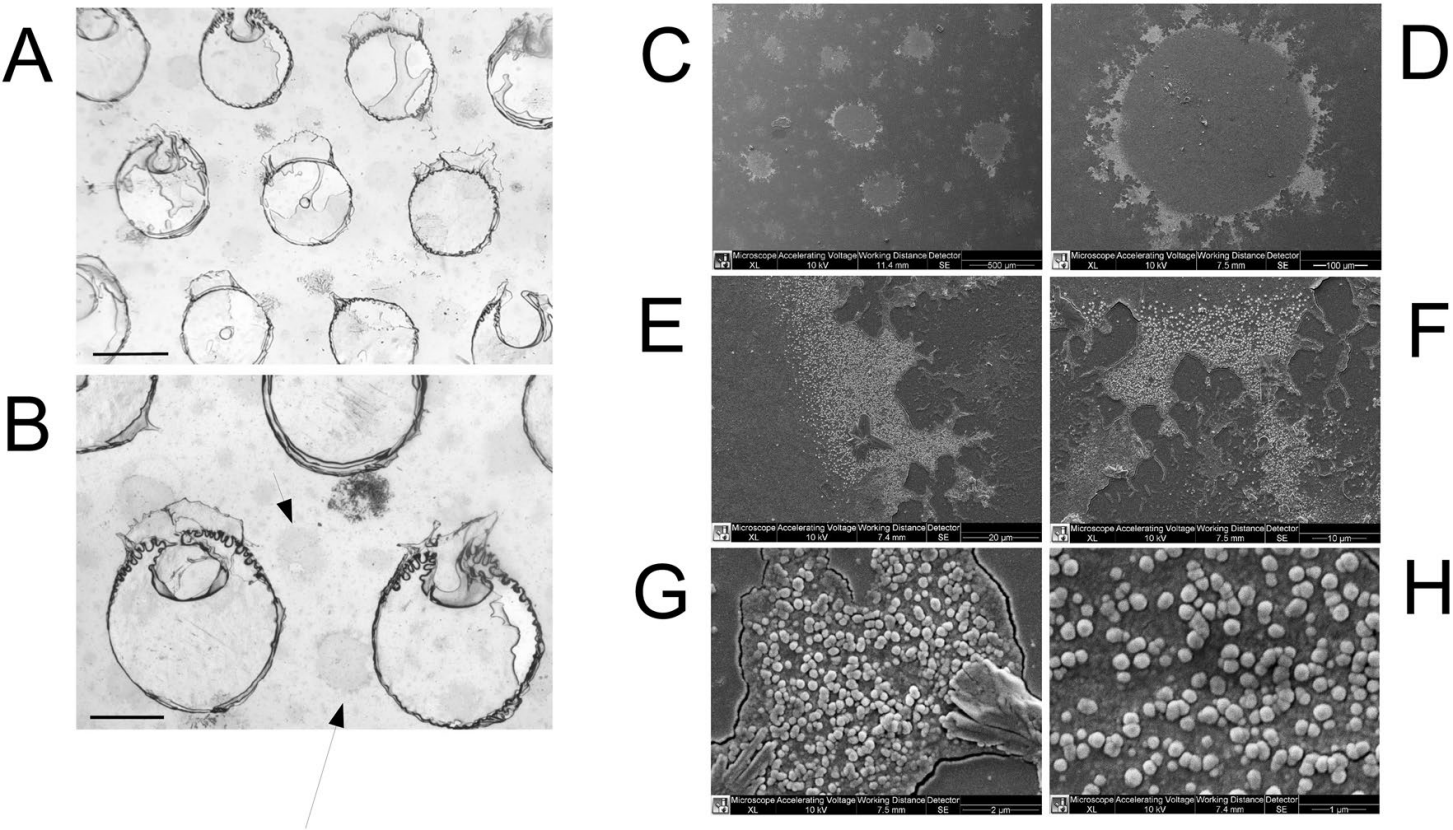
Optical (**A**, **B**) and Scanning Electronic Microscopy (SEM) (**C**–**H**) imaging of the WIWELL TiO_2_–Ag–NP photocatalytic surface. (**A**) Porose structure of the film (width bar = 1 mm); (**B**) Structure of the photocatalytic film with photocatalytic macro-spots (arrows) (width bar = 0.5 mm); (**C**) SEM of the photocatalytic macro-spots (500 μm), with an exemplificative imaging sample (**D**) showing the fractal-like dispersion area at the boundary (100 μm). (**E**) Focus at the boundary at 20 μm and (**F**) 10 μm, showing the photocatalytic complex; (**G**) Nano-imaging of the TiO_2_–Ag–NP complex at 2 μm and (**H**) 1 μm.

An optical and SEM imaging of the TiO_2_–Ag–NP photocatalytic film are shown in Fig. 9.

## Discussion

### Photocatalytic function of the WIWELL TiO_2_–Ag–NP product

Titanium dioxide is an excellent candidate for the production of photocatalytic devices, yet recent data reported that TiO_2_ by alone has a low solar conversion efficiency, approaching to 4%^51^. This efficiency can be improved by using anatase titanium dioxide doped with silver nanoparticles to obtain a one-dimensional composite material. TiO_2_ acts as an *n-*type semiconductor in the photocatalytic film. The use of silver (Ag) in the photocatalytic film has been preferred to other metals (Pt, Pd, W, Re, Ru, Os, Ir), normally adopted, as well as Ag, to enhance the Schottky barrier between interfaces with TiO_2_, simply because much it is currently cheaper and more available than other ≥ 1,000 USD priced elements (Ag = 23.62 USD/kg). Silver is a less expensive metal used to enhance the potential energy barrier for those electrons formed at a metal–semiconductor junction, known as Schottky barrier.

It is well known that upon UV light irradiation, for example UV components from direct solar light, only the semiconductor, i.e., TiO_2_, is excited. In this circumstance, to improve the photocatalytic potential of a product with TiO_2_, doping the photocatalytic material with metal nanoparticles, allows these latter to act as a kind of sink for electrons induced by light photons, via the Schottky barrier, a condition that prolongs the lifespan of these electrons, by reducing the recombination rate^52,53^.

Once a light source reached the WIWELL TiO_2_–Ag–NP photocatalytic film, a photon transfers a valence electron from the valence orbital of TiO_2_ (in the valence band VB) to the conductivity zone (CB), leaving an empty orbital in the VB (Eq. 1). As anatase TiO_2_ has a threshold bandwidth of 3.2 eV, any photon energy over this threshold will excite electrons from the 2p orbitals of oxygen (VB) to the 3d orbitals of titanium (CB):

1 $$ {\text{TiO}}_{2} + {\text{h}}\upnu \to {\text{h}}^{ + } + {\text{e}}^{ - } $$

where hν is a quantum of light (photon).

Actually, titanium oxide has a rather wide band gap, ranging from 3.20 eV (384 nm) in the crystalline form of rutile to 3.02 eV (410 nm) in anatase, and this is a limit, because it would allow all titanium dioxide to absorb only 4% of the solar light spectrum. However, the great advantage of TiO_2_ is its high semi-conducting property, which therefore makes it an excellent photo-catalyst. The aforementioned electron transfer, leaves holes in the top of the VB, so generating an electronic potential difference, separated by the space-charge layer, assessed by the presence of the metal nanoparticle (such as Ag–NP). Holes (positive charges) react with water molecules, generating hydroxyl radicals (·OH^-^) and hydroxyl ions (OH^-^), extremely reactive and oxidizing:

2 $$ {\text{h}}^{ + } + {\text{H}}_{2} {\text{O}} \to \cdot {\text{OH}} + {\text{H}}^{ + } $$

3 $$ {\text{h}} + {\text{OH}}^{ - } \to \cdot {\text{OH}} $$

The hydroxyl radical (·OH^-^) is formed by the surface water layer on the WIWELL TiO_2_–Ag–NP photocatalytic film but also oxygen can be, though in a lesser extent, a source of extremely reactive radicals, such as the superoxide anion (·O_2_^−^):

4 $$ {\text{e}}^{ - } + {\text{O}}_{2} \to {\text{O}}_{2}^{ - } $$

The superoxide anion can form perhydroxyl radicals (HOO·)

5 $$ \cdot{\text{O}}_{2}^{ - } + {\text{H}}^{ + } \to {\text{HOO}}\cdot $$

which alongside with hydroxyl and superoxide radicals, oxidize and damage bacterial membranes and viral capsids. Furthermore, these species self-generate other radicals, via oxygen and hydrogen peroxide such as:

6 $$ 2{\text{HOO}} \cdot \to {\text{H}}_{2} {\text{O}}_{2} + {\text{O}}_{2} $$

7 $$ {\text{e}}^{ - } + {\text{H}}_{2} {\text{O}}_{2} \to {\text{OH}}\cdot + {\text{OH}}^{ - } $$

The anti-microbial activity of the WIWELL TiO_2_–Ag–NP film is warranted by the oxidative, peroxidative and disrupting action of hydroxyl, superoxide and perhydroxide radicals on microbes’ molecular structures.

When the WIWELL TiO_2_–Ag–NP adhesive film is in a complete darkness (usually illuminance is ≤ 15 lx), the TiO_2_ photocatalytic activity slopes down significantly, accounting only on residual H_2_O_2_ and reactive oxygen species, so weakening and/or exhausting its photocatalysis-mediated organic degradation in a time dependent way, usually within 10–30 min^54^.

### Performance of the WIWELL TiO_2_–Ag–NP product

The application of our TiO_2_-Ag nano doped photocatalytic mixture (WIWELL) on a polyvinyl plastic adhesive film (WIGLASS), we collectively identified as TiO_2_–Ag–NP adhesive film, has generated a nanotechnological photocatalyst able to exert a biocidal activity towards airborne microbial particles, usually spread all over the indoor space, so reducing the microbial viability and promote the cleaning/sanitizing the environment. This study represents a further assessment of previous outcomes obtained with using our TiO_2_ photocatalysts in public transportation^20^, but, still represents a pilot study, as the activity of photocatalytic membranes for indoor air cleaning and sanitization via a low expenditure, feasible and straightforward approach, may be quoted as a real novelty in the field, if we except only few reviewed evidences, yet mainly reporting the use of photocatalytic membrane reactors on wastewater and water sanitization^15,24,55^. In this manuscript we have described the ability of a TiO_2_–Ag–NP to cleanse the different indoor spaces we investigated by dropping down the microbial presence even to values below 35 CFU/m^3^ and RLU ≤ 20, which are considered as biological markers of an extremely high purity of the indoor air climate. This study represents a novelty in the field of feasible, easy to handle and cost-effective thin film of TiO_2_–Ag–NP photocatalytic membranes for microbial cleansing in indoor spaces. In-progress studies from ours are suggesting so far that the ability of these devices to maintain a cleansed and healthy indoor living space, is long lasting, widely exceeding also the evaluation periods here described and extending for several months (yet not published data).

The use of Ag–NP, according to recent XRD data, enhances (using doping approaches) the stability of the titanium anatase phase, increasing also the stability property of the Ag–NP in the material^56^.

Despite the aforementioned values were obtained in standardized conditions and far from overcrowded indoor spaces, yet the TiO_2_–Ag–NP reported the capability to drop down microbial microparticles from the indoor air volume for at least 70% very early and by the first 6 h, according to our estimation in commonly lived, crowded and not standardized conditions. The WIWELL TiO_2_–Ag–NP adhesive film reaches the best performance (≤ 20 RLU) within 60 min from a 0 point (RLU ≥ 250) under daylight in a standardized, constant and closed indoor space. This should suggest that a healthy, clean and even purified indoor microenvironment can be easily reached and maintained throughout a working or living day in a stable ventilated and thermoregulated condition. Photocatalyst’s breakdown of microbial pollution goes ahead to reach a condition very close with a sanitized micro-environment, standing constant light emission and moderate, controlled inner ventilation^19,57^. The extremely ease in handling, the feasibility of any cleansing process by simply attaching the TiO_2_–Ag–NP on a vertical wall or any other enlightened surface^20^, low-cost and less time consuming technology, make this a real straightforward approach to abruptly reduce microbial pollutants and leave a healthy and comfortable living space, particularly for frail people.

This research is pivotal to assess the actual effectiveness of these membrane photocatalytic reactors directly in indoor spaces usually crowded by humans.

### Innovations and limitations of the study. Pros and cons

Aside from material and technical informations protected by trade secrets, including the plastic component and the methodology to build up the TiO_2_–Ag–NP photocatalytic film, the main innovation of WIWELL TiO_2_–Ag–NP photocatalytic product is its feasibility and ease to use, associated with an excellent performance in reducing microbial pollution in any indoor environment by simply applying the adhesive photocatalytic film on an enlightened surface^20,58^ (Fig. 1).

The selection of TiO_2_ as the major photocatalyst was due to the excellent optical and electronic properties of the material, its high chemical stability, no-toxicity, very low cost and environmental friendliness^59^. Major advantages in using TiO_2_ respect to other cheap semiconductors, such as bivalent zinc oxide (ZnO) or the tetravalent tin oxide (SnO_2_), come from the evidence that, using the TiO_2_ photocatalyst, a higher photodegradation rate (0.34/h) of Procion Red MX-5B was observed, respect to ZnO (0.25/h) and SnO_2_ (0/h)^60^. However, main disadvantages in using TiO_2_ as a photocatalyst is its wide band gap and a relatively short recombination time of electron carriers, a drawback which somehow limits the application of TiO_2_ by alone in the visible light region so affecting the photocatalytic efficiency. Doping with metal nanoparticles, such as Cu, Mg and Ni, in a nanosized metal/semiconductor heterojunction, resulted in an increasing photocatalytic performance (evaluated as the kinetics of photodecomposition of an organic test molecule) as well as Ag (Cu 0.6 wt%, *k* = 0.022/min, Mg 0.9 wt%, *k* = 0.019/min, Ni 0.5 wt%, *k* = 0.013/min)^61^, as the metal nanoparticle/semiconductor heterojunction extends the separation time between *e−* and *h* + and increases therefore the charge transfer rate. In this context, Ag (*k* = from 0.049 to 0.111/min in the range 0.05–0.174 wt%)^61,62^ was preferred to Cu and other metals.

The technological innovation of the WIWELL TiO_2_–Ag–NP adhesive film can be mainly referred to its environmental sustainability, the device is easy to be applied in any indoor space, lasts several months as it is far from any contact, can be easily purchased and used and moreover it reduces the microbial pollution without recurring to expensive and burdensome revisions of the indoor building to include mechanical ventilation or other purification electro-mechanical devices.

Furthermore, as the WIWELL TiO_2_–Ag–NP adhesive film has a microporous structure, it accelerates, via an adsorptive-membrane retention, the organic degradation mediated by photocatalysis and promotes the presence of water-caused micro- and nanobubbles due to the H_2_O supramolecular structure and tensioactivity^63^.

The results herein described are encouraging but with some limitation.

First, the number of samples should be further improved and their elaboration much more focused to draw important and sound data about the microbial turnover in a treated indoor space. Second, the existence of outliers and data with some difficulty in being reiterated depends on the complexity of “in field” research, therefore an improved standardization of methods and approach is paramount, for the next research of ours.

Finally, this study represents a forerunner in this research field somehow. Therefore, further insights are needed.

## Conclusions

A commercial TiO_2_–Ag nano doped photocatalytic mixture (WIWELL) on a polyvinyl plastic film, here indicated as TiO_2_–Ag–NP membrane, exhibited the ability to reduce drastically the microbial pollution in different kinds of indoor spaces by simply applying this photocatalytic device inside the building room. The described TiO_2_–Ag–NP adhesive film may be a promising tool to cleanse air from microbial and fungal contamination within few hours by simply attaching the photocatalyst on a wall, in an easy to handle, feasible and cost-effective strategy.

## Data Availability

The datasets used and/or analysed during the current study available from the corresponding author on reasonable request. Images (photos) are original and provided by Luca Berto (Fig. 1) and by Paolo Bernardi (Fig. 9).
